# Initial experience with combined heart-liver transplantation in the Netherlands: Exploring the boundaries of isolated and combined transplantation

**DOI:** 10.1007/s12471-025-01969-w

**Published:** 2025-07-22

**Authors:** Ryan E. Accord, Frans J. C. Cuperus, Elke Hoendermis, Massimo Mariani, Gianclaudio Mecozzi, Maarten W. Nijkamp, Vincent E. de Meijer, Joost M. Klaase, Hans Blokzijl, Meine H. Fernhout, Koen M. E. M. Reyntjens, Joost M. A. A. van der Maaten, Marije Smit, J. Droogh, Michiel E. Erasmus, Kevin Damman, Joost P. van Melle

**Affiliations:** 1 Department of Cardiothoracic Surgery, Centre for Congenital Heart Disease, University of Groningen, University Medical Centre Groningen, Groningen, The Netherlands; 2 Department of Gastroenterology and Hepatology, University of Groningen, University Medical Center Groningen, Groningen, The Netherlands; 3 Department of Cardiology, Centre for Congenital Heart Disease, University of Groningen, University Medical Centre Groningen, Groningen, The Netherlands; 4 Department of Cardiothoracic Surgery, University of Groningen, University Medical Centre Groningen, Groningen, The Netherlands; 5 Department of Hepato-Pancreato-Biliary Surgery and Liver Transplantation, University of Groningen, University Medical Centre Groningen, Groningen, The Netherlands; 6 Department of Anesthesiology, University of Groningen, University Medical Centre Groningen, Groningen, The Netherlands; 7 Department of Intensive Care, University of Groningen, University Medical Centre Groningen, Groningen, The Netherlands

**Keywords:** Congenital heart disease, Fontan failure, Fontan-associated liver disease, Heart transplantation, Heart-liver transplantation

## Abstract

Heart transplantation is considered as the ultimate treatment for patients with advanced heart failure (HF). Chronic HF is associated with hepatic congestion and reduced cardiac output, which can lead to progressive liver disease. This issue is particularly relevant in patients with congenital heart disease, especially those with a single functional ventricle managed through Fontan-type surgery. The presence of advanced liver disease may contraindicate isolated heart transplantation and thus require consideration of combined heart-liver transplantation (CHLT). However, consensus criteria for CHLT have not yet been established. To illustrate the clinical and scientific discussions on this topic, we present the clinical course of two patients with a Fontan circulation who were evaluated for CHLT and discuss decision-making factors based on a review of current literature. We conclude that establishing a CHLT program represents a promising therapeutic pathway for patients in the Netherlands with advanced HF and concomitant liver disease. Both isolated heart transplantation and CHLT are viable treatment approaches for carefully selected patients with HF and liver disease. However, early identification of potential candidates and timely referral for a comprehensive evaluation are essential for the effective management of this high-risk patient group.

## Introduction

Heart transplantation represents the ultimate therapeutic intervention for patients with advanced heart failure (HF). However, a subset of these patients develops advanced liver disease, which may affect eligibility for isolated heart transplantation. This issue is particularly relevant in patients with congenital heart disease (CHD), such as those with a functionally univentricular heart. For these patients, a series of surgical procedures will establish what is known as a Fontan circulation, first described in the early 1970s [1, 2]. This circulation redirects desaturated systemic venous blood directly into the pulmonary circulation, bypassing the functionally single ventricle. The Fontan circulation aims to prevent intracardiac shunting, oxygen desaturation, and ventricular volume overload. However, the absence of a subpulmonary ventriclealso leads to chronically elevated systemic venous vascular pressure and a decreased cardiac output [3]. Although optimised management has increased the life expectancy in patients with a Fontan circulation, complications such as arrhythmias, ventricular dysfunction, and extra-cardiac organ involvement inevitably lead to a premature decline in cardiovascular performance (‘failing Fontan’) and reduced survival, even in the best-managed patients [4, 5]. The Australia/New Zealand Fontan Registry reports a 62% survival rate at 35 years, while a 20-year survival of 68% has been reported in Dutch/Belgian patients with a single-ventricle atrioventricular septal defect [6, 7]. In contrast to mainstream cardiology, conventional HF therapies offer little to no benefit for patients with a failing Fontan circulation. Additionally, the use of mechanical circulatory support is limited in this population, as anatomical variations and the absence of a subpulmonary ventricle complicate the application of conventional devices designed for biventricular physiology [8, 9]. As a result, heart transplantation remains the only definitive treatment for patients with a failing Fontan circulation.

**Fig. 1:**
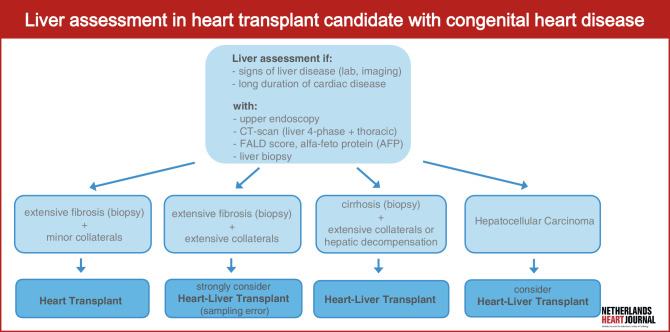
Infographic

Chronic systemic venous congestion, decreased cardiac output, and hypoxia result in the development of Fontan-associated liver disease (FALD), which encompasses all hepatic alterations secondary to the Fontan circulation and is thus present, to varying degrees, in every Fontan patient [10]. FALD can progress from hepatic fibrosis to advanced liver disease with portal hypertension (varices, portosystemic collaterals, ascites, and/or splenomegaly) and is associated with an increased risk of hepatocellular carcinoma (HCC) [11, 12]. Advanced FALD, recently defined as FALD with portal hypertension [10], may potentially contra-indicate isolated heart transplantation and necessitate consideration of combined heart-liver transplantation (CHLT) (Fig. 1). The impact of FALD on patient survival remains poorly defined. Although FALD-specific scoring systems may predict prognosis to some extent, consensus criteria for selecting candidates for CHLT versus isolated heart transplantation are currently lacking. Screening for CHLT is complex due to the absence of evidence-based protocols, center-based differences in resources and clinical practice, and patient heterogeneity, and requires a multidisciplinary approach. To illustrate this complexity, we present two illustrative clinical cases; one managed with CHLT and the other with isolated heart transplant, and provide a review of the current literature on CHLT. Through this discussion, we aim to elucidate the clinical, ethical, and logistical challenges in managing this unique patient population.

## Case 1

**Fig. 2:**
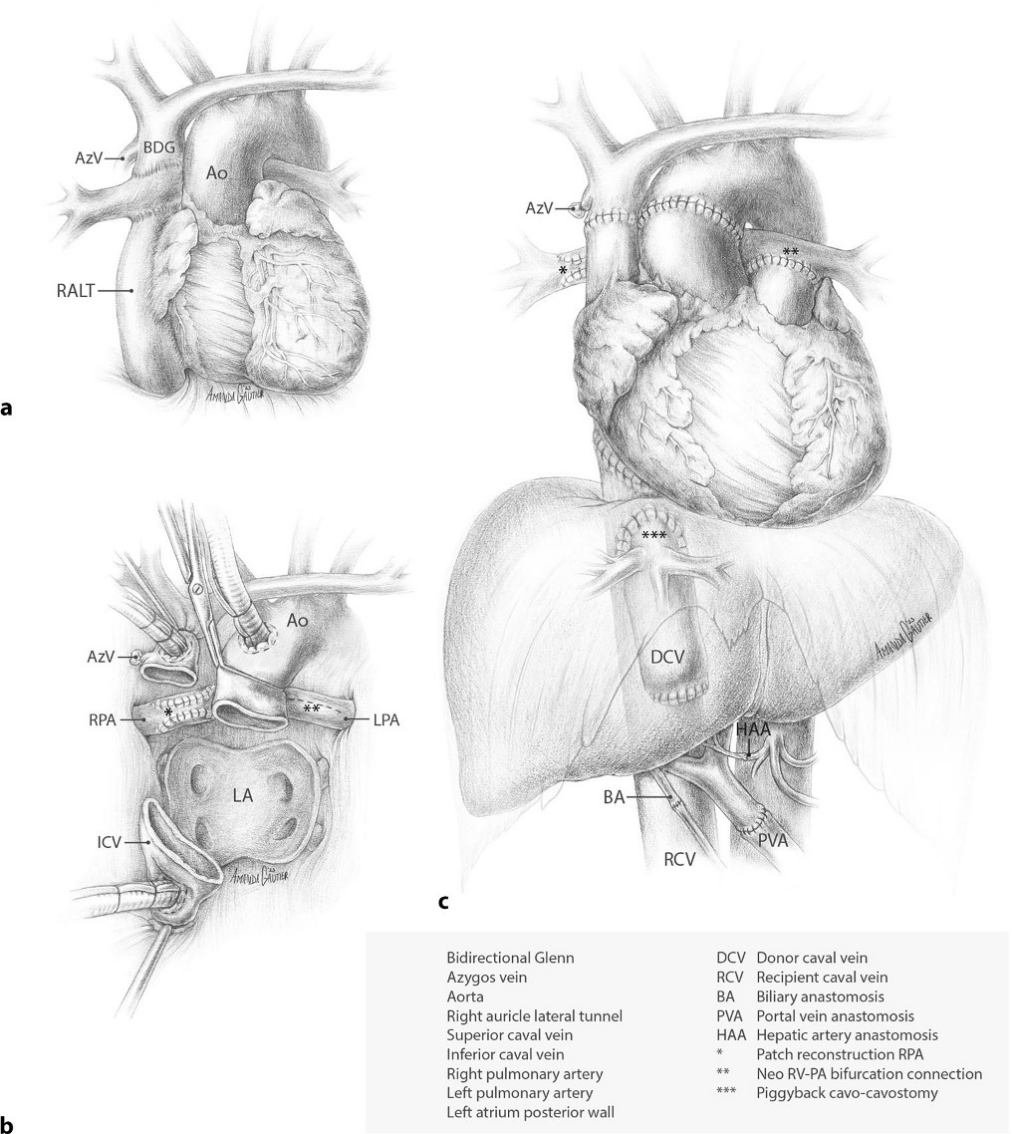
Panel **a** shows the anatomic situation in patient 1 with lateral tunnel Fontan circulation. Panel **b** pericardial view after completed cardiectomy and patch reconstruction of the right pulmonary artery in preparation for orthotopic heart transplantation. Panel **c** depicts the end result after a combined heart-liver transplantation

The patient’s diagnosis at birth was a double inlet left ventricle with a hypoplastic right ventricle, ventricular septum defect with straddling of the tricuspid valve, transposition of the great arteries, and a persistent ductus arteriosus. A series of palliative procedures followed, eventually leading to the construction of a bidirectional Glenn anastomosis (connection of superior caval vein to the pulmonary artery (PA)), in combination with a lateral tunnel (inferior caval vein to PA). The main PA was disconnected from the PA confluence and oversewn (Fig. 2a). Three additional cardiac operations followed, aiming to optimise the Fontan circulation: implantation of an epicardial pacemaker system for sick sinus syndrome; resection of a subaortic stenosis and patch closure of the tricuspid valve to address tricuspid regurgitation; and, most recently, correction of residual tricuspid leakage with enlargement of the VSD, which is part of the subaortic outflow tract. However, the diagnostic criteria for a failing Fontan were met in 2016, including atrial arrhythmia despite medication and ablations, ascites, deteriorating functional capacity, and decreased quality of life. Eventually, this required repeated hospitalisations in 2021–2022.

Due to suspected advanced FALD, the patient underwent CHLT screening. Upper gastrointestinal endoscopy demonstrated medium-sized esophageal varices. An abdominal CT scan demonstrated signs of portal hypertension, including ascites, portosystemic collaterals, umbilical vein recanalization, and splenomegaly. Finally, a liver biopsy demonstrated cirrhosis (congestive hepatic fibrosis score 4). The FALD score was 3 (1 point each for the presence of cirrhosis, esophageal varices, splenomegaly, ≥ 2 paracenteses) [13]. After an extensive screening period and development of a comprehensive perioperative plan, the patient was listed for CHLT in September 2022.

### Organ procurement and preservation

The heart and liver were retrieved (donation after brain death) by separate thoracic and liver procurement teams. Considering the recipient’s anatomy, the heart team ensured sufficient length of superior caval vein (SCV) and AP bifurcation. Liver procurement was performed as usual en bloc with the pancreas followed by back-table separation, including an additional portal flush with preservation solution. After procurement, the heart and liver were packed on ice separately. Upon arrival at our center, the liver was preserved using *ex situ* hypothermic (10 °C) oxygenated machine perfusion (LiverAssist, XVIVO, Groningen, the Netherlands) to avoid prolonged cold ischemia time, as described previously [14–16].

### Surgical procedure

Following chest re-entry and uncomplicated dissection of the heart, the cardiopulmonary bypass commenced. Recipient cardiectomy and detachment of both cavo-pulmonary connections from the right PA necessitated the reconstruction of these PA defects with bovine pericardial patches (Fig. 2b). The donor heart was introduced into the pericardial cavity, and intermittent retrograde administration of oxygenated cold blood cardioplegia via the coronary sinus was maintained during implantation. The left atrial, inferior vena cava (IVC)-to-right atrium, aortic, and the PA to PA-confluence anastomoses were completed consecutively. After finalising the SVC anastomosis, the aortic cross-clamp was removed, initiating reperfusion of the donor heart (ischaemia time 3:48 h), followed by uneventful weaning from cardiopulmonary bypass. Cardiac contractility, assessed by haemodynamic parameters and intraoperative transesophageal echocardiography, was satisfactory. After chest closure, a bilateral subcostal incision was made for the subsequent liver transplantation.

After an uncomplicated recipient hepatectomy, the donor liver was disconnected from the perfusion machine (4:41 h of static cold ischaemia time and an additional 6:48 h of hypothermic-oxygenated machine perfusion preservation). The liver graft was implanted using the standard piggyback technique and partial IVC clamping. Graft reperfusion (warm ischaemia time 36 min) was achieved via the portal vein, followed by end-to-end arterial and duct-to-duct biliary anastomosis. During reperfusion and the postoperative period, the recipient remained hemodynamically stable with primarily vasopressor therapy and nitric oxide ventilation without post-operative extracorporeal life support. Total liver preservation time was 12:58 h. The result after CHLT is depicted in Fig. 2c.

### Post-operative course

The postoperative course was complicated by hepatic artery thrombosis (HAT) on days 1 and 11, which required surgical revision with a patent artery as a result. The remaining intensive care unit (ICU) stay was marked by respiratory insufficiency (likely due to ICU-acquired weakness, transient diaphragm paresis, and fluid retention) as well as acute kidney injury, necessitating short-term hemodialysis. The patient could be discharged from the hospital on day 58 and currently remains in good clinical condition 2 years after transplantation, without signs of rejection.

## Case 2

The second patient was diagnosed with tricuspid atresia at birth. After initial palliation with a Waterson shunt (aorto-to-right PA connection to provide adequate pulmonary flow), a Fontan procedure with an atriopulmonary connection (right atrial appendage-to-PA) was performed. The following decades were characterised by recurrent atrial arrhythmias, treated medically and with repeated electrical cardioversions, multiple endocardial ablation procedures, and eventually epicardial pacemaker implantation and His-bundle ablation, and the diagnosis of failing Fontan was established based on frequent arrhythmias and reduced exercise tolerance. The risk-benefit ratio of extracardiac tunnel conversion was considered unfavourable, and the patient was screened for CHLT due to suspicion of advanced FALD. Upper gastrointestinal endoscopy demonstrated small-sized esophageal varices. Abdominal CT scans (in 2021 and 2023) demonstrated a small amount of ascites, umbilical vein recanalisation without other portosystemic collaterals, and no splenomegaly. Finally, liver biopsies in 2021 and 2023 demonstrated septal and sinusoidal fibrosis without progression to cirrhosis (congestive hepatic fibrosis score 2). The FALD score was 2. Although the patient showed mild signs of portal hypertension, we decided to list the patient for isolated heart transplantation. The patient received a donor heart (donation after brain death) and underwent an uncomplicated heart transplantation as described in the first patient. Post-operatively, a decrease in right ventricular function was successfully treated with milrinone and diuretics. The postoperative course was uncomplicated, and the patient was discharged on day 18. Five months after discharge, the patient was admitted after an out-of-hospital cardiac arrest. Evaluation revealed pre-existing coronary artery disease in the donor heart (no coronary angiogram was performed per protocol, due to the young age of the donor). One year after transplantation, no liver-related adverse events were reported. Liver elastography (Fibroscan) showed a significant decrease in liver stiffness (from 33 kPa before heart transplantation to 7 kPa one year thereafter). The patient remains in good clinical condition after 18 months of follow-up.

### Indications for CHLT

Isolated liver transplantation is not recommended in patients with advanced FALD due to the challenging perioperative management resulting in poor outcomes [17]. While liver transplantation is an important treatment option for HCC in cirrhosis, the role of CHLT is less established in Fontan-related HCC due to limited data. Consequently, the benefits of CHLT versus locoregional treatment require case-by-case evaluation [10]. CHLT should be considered in patients with advanced FALD, recently defined as patients with signs of portal hypertension such as varices, ascites, portosystemic collaterals, and ascites secondary to FALD [10]. In a retrospective cohort of 73 patients with a Fontan circulation, portal hypertension as determined by a VAST score of ≥ 2 (1 point each for the presence of oesophageal Varices; Ascites, Splenomegaly, Thrombocytopenia) was associated with an increased risk of death, HCC and heart transplantation [18]. However, there is insufficient evidence to routinely recommend CHLT in all patients with a failing Fontan and advanced FALD. Several studies have found no difference in survival between cirrhotic and non-cirrhotic patients with a Fontan circulation after isolated heart transplantation [19–21]. Other studies, have demonstrated that the MELD-XI score, an adapted MELD score for FALD that excludes international normalized ratio, can predict mortality after isolated heart transplant [22]. The largest retrospective cohort comparing CHLT and isolated heart transplantation in adult Fontan (40 CHLT and 91 isolated heart transplantation) from 15 transplant centers across the United States and Canada demonstrated that a FALD-specific score of ≥ 2 was associated with superior survival in adult CHLT patients compared with heart-only recipients [13]. Although these data suggest a survival benefit for CHLT in advanced FALD, the retrospective design of all the above-mentioned studies inherently confers residual confounding and bias. Consequently, prospective validation is warranted to understand in which patients CHLT is superior to single heart transplantation [17, 23].

When a patient with a Fontan circulation requires a heart transplantation, the severity of FALD must be assessed. However, FALD severity is not easily defined. Liver tests remain near normal, even in advanced FALD. Esophageal varices can occur in the absence of severe liver fibrosis due to veno-venous communications of the upper venous plexus [10]. Liver stiffness measurement (LSM) does not reflect hepatic fibrosis accurately, due to hepatic congestion. Abdominal imaging typically demonstrates irregular and bulging liver contours, atrophy of the right lobe and a nodular aspect of the liver due to hepatic congestion and does not reflect cirrhosis [24, 25]. Nevertheless, contrast-enhanced cross-sectional imaging (CT and/or MRI) is important both in determining the extent of portosystemic collaterals as a sign of portal hypertension, as well as the presence of HCC. Finally, a liver biopsy, although essential in evaluating patients for CHLT, may underestimate fibrosis due to sampling error [10, 26]. Altogether, most clinical findings that assess the severity of liver disease and cirrhosis are unreliable in patients with a Fontan circulation, underlining the challenge in assessing FALD severity. The impact of FALD on patient survival is not well-defined, and consensus criteria for CHLT are currently lacking. In addition, the model for end-stage liver disease (MELD) score, which determines priority for liver transplantation, is based on laboratory values that remain relatively normal in FALD, and may not accurately assess FALD disease severity. Currently, no specific liver disease scoring system can accurately predict survival in Fontan patients after transplantation, although the FALD-score seems to hold some promise.

## Future perspectives of CHLT in the Netherlands

**Fig. 3:**
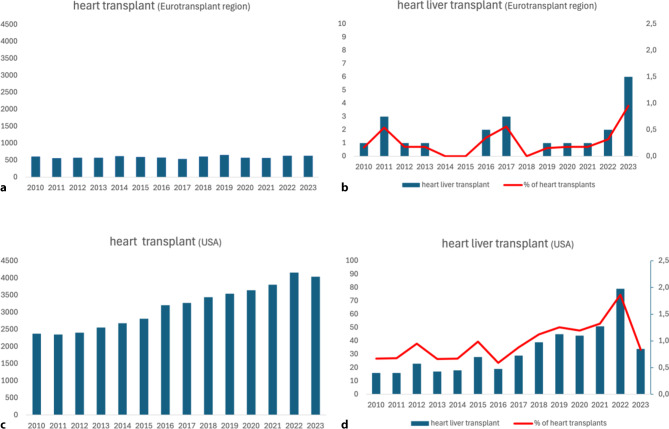
Panel **a** total number of heart transplants in the Eurotransplant region (Austria, Belgium, Croatia, Germany, Hungary, Luxembourg, the Netherlands and Slovenia). Panel **b** total number of heart liver transplants in the Eurotransplant region in actual numbers (bars) and as a percentage of all heart transplants (line). Data obtained from Eurotransplant. Panel **c** total number of heart transplants in the in United States. Panel **d** total number of heart liver transplants in the Eurotransplant region in actual numbers (bars) and as a percentage of all heart transplants (line). Please note that the y‑axis scale differs from that in Panel **b**. Data obtained from the Organ Procurement and Transplantation Network

CHLT is a complex surgical procedure that remains infrequently performed in Europe. Between 2010 and 2023, only 24 CHLTs (0.27% of heart transplants) were performed in the Eurotransplant region, whereas 5 CHLT were performed in the UK (0.35%) from 2011 to 2021 [27]. In contrast, the United States recorded 458 CHLT procedures from 2011–2021, accounting for 1% of total heart transplants (Fig. 3). The favourable survival rates (1-year survival of 89%, 5‑year survival of 84%) [13] and decreased incidence of graft rejection in CHLT have likely contributed to the increase in CHLT in the United States (Fig. 3; [28, 29]). Although infrequently performed, there is an increasing demand for CHLT due to the growing population of patients with complex CHD surviving into adulthood. CHD has now become the most frequent indication for CHLT, surpassing non-congenital heart disease [28]. One of the major benefits of a CHLT program is the opportunity to evaluate patients with suspected advanced liver disease who would normally not be considered for isolated heart transplantation. Assessment by a dedicated multidisciplinary team facilitates the appropriate selection of patients requiring CHLT and those for whom heart transplantation alone is still possible, despite concomitant liver disease.

The presence of a CHLT program is particularly important for patients with a Fontan circulation. The number of Fontan patients is estimated at approximately 66 per 1 million inhabitants, based on prevalence data in 11 countries, corresponding to ~1,200 patients in the Netherlands. This number is expected to rise to 79 patients per million by 2030 [30]. The vast majority of these patients survive into adulthood but eventually develop progressive HF and multiorgan dysfunction. Adult CHD cardiologists are increasingly confronted with detrimental long-term sequelae of the Fontan circulation, including fatigue, arrhythmias, decreased cardiovascular performance, and end-organ complications such as protein-losing enteropathy and FALD. While transplantation remains the only definitive treatment, it is challenging due to the heterogeneity in underlying cardiac anomalies and technical considerations including suitability of anastomoses, previous sternotomies, lack of vascular access, bleeding-risk due to collaterals, anesthetic management, human leukocyte antigen allosensitisation, and malnutrition. The optimal timing for referral, screening, and listing of failing Fontan patients is key, but subject to debate since conventional HT indications, often but not always based on ventricular dysfunction, are less applicable in this population where ~40% maintain preserved ventricular function at referral [31]. Instead, the mode of failure is more diverse and includes both cardiac (e.g. arrythmias) and non-cardiac (e.g. protein-losing enteropathy, FALD) complications, as described above.

## Centre experience

**Fig. 4:**
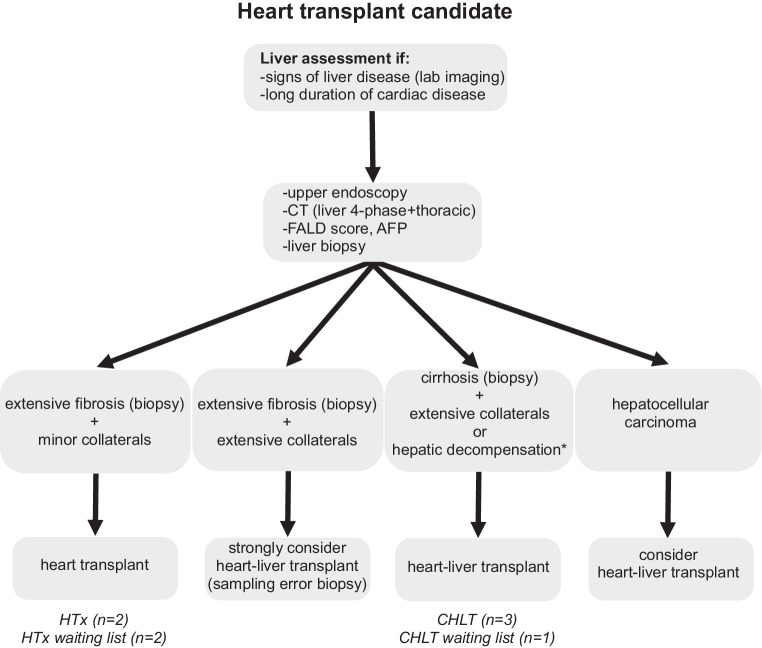
Flowchart for liver assessment in patients with congenital heart disease/Fontan physiology to be considered for isolated heart transplant or combined heart-liver transplant. Text in *Italic* includes evaluated patients who received/were enlisted for combined heart-liver transplant (CHLT) or isolated heart transplant (HTx). Patients without extensive fibrosis or collaterals are not included in this flowchart. *Hepatic decompensation includes severe synthetic dysfunction of the liver, hepatic ascites (low protein, high liver-ascites albumin gradient), hepatic encephalopathy, and recurrent portal hypertensive bleeding. *FALD* Fontan Associated Liver Disease, *AFP* alpha-fetoprotein

At our centre, all patients with failing Fontan who require heart transplantation and show evidence of coexisting liver disease, based on laboratory or imaging findings, are referred to transplant hepatology (Fig. 4). These patients undergo additional liver transplant evaluation consisting of laboratory tests, upper endoscopy to detect esophageal varices, contrast-enhanced cross-sectional imaging to visualise portosystemic collaterals and screen for HCC, and a liver biopsy. Patients with biopsy-proven cirrhosis and/or significant portal hypertension are considered for CHLT. Contraindications for CHLT are listed in Tab. 1. Both patients had advanced FALD, albeit to a different degree, but only the first patient was listed for CHLT due to the presence of significant portal hypertension with medium-sized varices, portosystemic collaterals, and biopsy-proven cirrhosis. This resulted in a FALD score of 3, associated with superior survival for CHLT [13]. The second patient only had mild signs of portal hypertension and septal fibrosis without progression to cirrhosis between 2021 and 2023. The FALD score in this patient was 2, which would still imply a survival benefit for CHLT compared to isolated heart transplantation [13]. However, the continued absence of cirrhosis led to the decision to list for isolated heart transplantation. Both patients were transplanted with satisfactory outcomes, highlighting the benefits of the CHLT program. Histopathological liver graft findings were consistent with pre-transplant biopsies in both patients. Interestingly, LSM of the second patient decreased to near-normal values one year after heart transplantation despite a cirrhotic aspect of the liver on cross-sectional imaging. The decrease in LSM might reflect not only a decrease in hepatic congestion but also a regression of biopsy-proven liver fibrosis after successful cardiac transplantation, as reported previously [32].

**Table 1:** Contra-indications for Combined Heart Liver Transplantation

Contra-indications	Relative contra-indications
Age above 70 years	Age above 65 years
Treatment-resistant (systemic) extrahepatic infections	Active infections
Multi-organ failure (other than heart & liver)	Chronic hepatitis B or hepatitis C
Irreversible pulmonary vascular resistance (above 400 dynes · s/cm^5^)	HIV-seropositivity
Transpulmonary gradient > 15 mm Hg	HLA allosensitization
Malignancy in the past 5 years (other than skin or liver malignancies)	Body Mass Index above 35 kg/m^2^
Irreversible brain damage accompanied by severe disability	Expectation of exceedance of maximal organ ischemia (e.g. due to expected surgical complexity)
Treatment-resistant psychiatric disorders	Diabetes mellitus with nephropathy, retinopathy and/or neuropathy
Active substance abuse	Peripheral (atherosclerotic) vascular disease
	Renal insufficiency expected to be non-reversible after transplantation (< eGFR 30 mL/min/1.73 m^2^)
	Hepatocellular carcinoma (only eligible if fulfilling national criteria for liver transplantation for hepatocellular carcinoma)
	Cholangiocarcinoma
	Psychosocial considerations (e.g. non-compliance)
	Patient without Dutch passport or residence permit

Based on our experience in ex-situ organ preservation techniques, a ‘heart-first’ sequential approach with hypothermic oxygenated machine liver perfusion was chosen, allowing prolonged graft preservation and patient stabilisation until the recipient is ready for liver implantation [14, 15]. En bloc CHLT does not allow stabilisation, but it may offer some surgical advantages and both approaches show comparable 1‑year survival rates [33–35].

The CHLT was complicated by HAT, an uncommon (< 1%) complication post-liver transplantation. We speculate that active correction of hemostasis after heart transplantation, a risk factor for HAT, might have contributed. So far, eight patients have been evaluated for CHLT in our centre; three have undergone CHLT, and one is awaiting CHLT; four were selected for heart transplantation alone, of whom two remain on the waiting list (Fig. 4).

Early referral of heart transplant candidates with evidence of liver disease is crucial due to the complex nature of the decision-making process in a high-risk population. Although an early isolated heart transplant might obviate the need for CHLT, this is a mere theoretical consideration, since cardiac dysfunction remains the primary indication for transplant evaluation, except for HCC. The decision to perform CHLT or isolated heart transplant should take both the outcome and the limited donor organ availability, particularly important in the Netherlands, into account.

## Conclusion

The development of a CHLT program provides a future perspective for patients in the Netherlands with advanced heart failure and concomitant liver disease. CHLT may be a feasible treatment option for selected patients with both heart and liver failure, especially those with CHD who were palliated with a Fontan procedure.
